# Safety and efficacy of atrial fibrillation ablation in kidney transplant patients

**DOI:** 10.1007/s10840-025-02006-x

**Published:** 2025-02-28

**Authors:** Ahmad Keelani, Lorenzo Bartoli, Alessio Gasperetti, Sorin Popescu, Marco Schiavone, Anna Traub, Huong-Lan Phan, Marcel Feher, Thomas Fink, Vanessa Sciacca, Martin Nitschke, Julia Vogler, Charlotte Eitel, Giovanni Forleo, Christian-H. Heeger, Roland R. Tilz

**Affiliations:** 1 Department of Rhythmology, University Hospital Schleswig-Holstein – Campus Lübeck, Lübeck, Germany; 2 Arrhythmia Section, Division of Cardiology, Heart Center, Zentralklinik Bad Berka, Bad Berka, Germany; 3 Institute of Cardiology, Sant’Orsola-Malpighi Hospital, IRCCS, Bologna, Italy; 4 Department of Experimental, Diagnostic and Specialty Medicine-DIMES, University of Bologna, Bologna, Italy; 5 Department of Cardiology, Luigi Sacco University Hospital, Milan, Italy; 6 Department of Cardiology, Johns Hopkins University, Baltimore, USA; 7 Department of Clinical Electrophysiology & Cardiac Pacing, Centro Cardiologico Monzino, IRCCS, Milan, Italy; 8 Department of Systems Medicine, University of Rome Tor Vergata, Rome, Italy; 9 Clinic for Electrophysiology, Herz- Und Diabeteszentrum NRW, Bad Oeynhausen, Germany; 10 Transplant Center, University Hospital Schleswig-Holstein – Campus Lübeck, Lübeck, Germany; 11 Department of Rhythmology, Asklepios Klinik Hamburg Altona, Hamburg, Germany; 12 German Center for Cardiovascular Research (DZHK), Partner Site Lübeck, Hamburg, Germany; 13 LANS Cardio, Hamburg, Germany

**Keywords:** Atrial fibrillation, Catheter ablation, Kidney transplant, Chronic kidney disease

## Abstract

**Introduction:**

Managing atrial fibrillation in kidney transplant patients poses a challenge for both nephrologists and cardiologists. Data regarding the safety and efficacy of catheter ablation in this patient’s cohort is scarce.

**Methods and results:**

In this two-center prospective study, we included all consecutive kidney transplant patients who underwent atrial fibrillation ablation between April 2017 and March 2022. A 1:3 propensity score matching created a control group of non-transplant AF patients undergoing ablation. We included 16 kidney transplant patients and 48 matched controls. Ablation was successful in all patients. The periprocedural complication rate (6.3% in the kidney transplant group vs. 6.3% in the control group, *p* value = 1) did not differ between the two groups. One transplant patient experienced graft dysfunction after a complication. At 18 months, AF recurrence-fee rates were 69% in the transplant group and 70.1% in controls (*p* = 0.95). By the last follow-up, all transplant patients had discontinued antiarrhythmic drugs, while 19.6% of the patients in the control group were treated with antiarrhythmic drugs (*p* = 0.09). Kidney function in the transplant group remained stable (eGFR 32 [23.8, 40.5] ml/min/1.73 m^2^ before vs. 34 [29.8, 38] ml/min/1.73 m^2^ at last follow up, *p* = 0.93).

**Conclusions:**

This study demonstrates that catheter ablation is a viable option for treating AF in kidney transplant patients, with comparable outcomes to non-transplanted individuals. Discontinuing antiarrhythmic drugs reduces drug interaction risks, but minimizing procedural complications remains critical to preserving graft function.

**Graphical Abstract:**

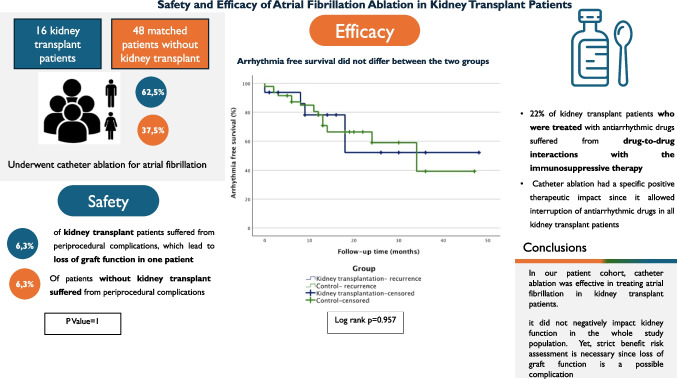

**Supplementary Information:**

The online version contains supplementary material available at 10.1007/s10840-025-02006-x.

## Introduction

Kidney transplant (KT) is the treatment of choice in patients with end-stage renal disease (ESRD). Compared to dialysis, KT leads to  improved survival, quality of life, and a better cardiovascular (CV) risk profile [1]. Nevertheless, due to a high burden of CV risk factors and the need for chronic immunosuppressive therapy, KT patients remain at higher risk for CV disease compared to the general population [2].

Atrial fibrillation (AF) is the most common arrhythmia in patients with chronic kidney disease (CKD) [3], and it is associated with progression to ESRD [4, 5]. AF affects 6% of patients undergoing KT [6] and increases the risk of post-transplant mortality, allograft loss, and stroke. KT does not decrease AF burden in ESRD patients, and AF prevalence reaches 7.3% at 3 years of post-transplantation [7]. Management of AF in KT patients represents a clinical challenge because of their high cardiovascular risk profile, adverse events of anticoagulation and antiarrhythmic therapy, and potential interactions between the latter and immunosuppressive drugs.

Catheter ablation (CA) has proved to be a safe and effective therapy for symptomatic AF patients [8], and in KT patients it may provide a solution to avoid the possible long-term side effects of antiarrhythmic drugs (AAD) and their interactions with immunosuppressive therapy.

Nonetheless, a periprocedural complication rate of 3.0–4,9% is non-negligible [9, 10], and this may be increased in KT patients, also due to a potential worsening of kidney function and allograft loss.

There is a paucity of data regarding AF ablation in KT patients. The aim of this study was to evaluate the safety and efficacy of this procedure in KT patients.

## Methods

### Study design and population

In this two-center prospective study, we included all consecutive KT patients who underwent AF ablation in two high volume European electrophysiology centers (University Hospital Schleswig–Holstein Campus Lübeck, Lübeck, Germany; Sacco University Hospital Milan, Milan, Italy) between April 2017 and March 2022.

We included symptomatic patients (EHRA score ≥ 2 or heart failure induced by the arrhythmia) with paroxysmal or persistent AF with an expected survival superior to 1 year.

 A matched control group (CG) of patients with symptomatic AF who underwent de novo PVI was selected using 1:3 propensity score matching.AF type (paroxysmal, persistent, or long-standing persistent), age, sex, and AF ablation technique were used as covariates. Patients of the CG were selected from the Lübeck ablation registry, which prospectively includes all patients receiving catheter ablation of atrial fibrillation and other cardiac arrhythmias at the Department of Rhythmology of the University Hospital Schleswig–Holstein – Campus Lübeck.

The study was conducted in compliance with the ethical standards outlined in the Declaration of Helsinki and approved by the local Ethics Committee (No. 15–347).

### Baseline data and kidney function evaluation

Baseline data included demographic features, type of AF (paroxysmal, persistent and long-standing persistent), CV risk factors, comorbidities, baseline anticoagulant therapy, echocardiographic, and laboratory parameters.

We estimated the glomerular filtration rate (eGFR) using the Chronic Kidney Disease Epidemiology Collaboration (CKD-EPI) formula. Subsequently, we used eGFR to classify patients’ CKD status using the Kidney Disease Improving Global Outcomes (KDIGO) guidelines [11]. Kidney function was assessed at enrollment, after the procedure and at each follow-up (FU) visit.

### Periprocedural and procedural management

Preprocedural assessment included laboratory tests and transesophageal echocardiography to exclude intracardiac thrombi. As regards oral anticoagulation (OAC) management, in patients on vitamin K antagonist (VKA), the target periprocedural international normalized ratio (INR) was 2.0–2.5. In patients on direct OAC (DOAC), we adopted a minimally interrupted OAC strategy, omitting the morning dose on the day of the procedure. We performed the procedures under deep sedation using a combination of midazolam, fentanyl, and a continuous infusion of propofol.

Ultrasonographic guidance was used to obtain femoral venous access. A diagnostic catheter (7F, Biosense Webster, Inc.) was inserted into the coronary sinus. We performed fluoroscopy-guided double transseptal puncture using an 8.5F transseptal sheath (SL1; St. Jude Medical, Inc.).

After the transseptal puncture, heparin was administered to achieve an activated clotting time (ACT) of 300 to 350 s. The PV ostia were identified using pulmonary vein (PV) angiography. The amount of contrast injected used for the procedure was carefully monitored and kept to a maximum of 30 ml in the KT group whenever possible. We used a no-contrast approach in selected patients with compromised kidney function, avoiding performing PV angiography. We used all the main available technologies to perform AF ablation (laser balloon ablation (LBA), cryoballoon (CB) and radiofrequency ablation (RFA)). A detailed description of each ablation technique is available in the supplementary materials of this article.

Finally, additional lesions (left atrial appendage (LAA) isolation, targeting of complex fractionated electrograms (CAFEs), additional ablation lines (roof, anterior, box and mitral isthmus lines)) were performed based on the operator’s judgement.

Pericardial effusion was ruled out immediately, 2 h, and on the first day after the procedure. We performed duplex sonography in patients with clinical suspicion of vascular access complications (pseudoaneurysm or arterio-venous fistula).

In the absence of vascular complications or a post-procedural pericardial effusion, we resumed OAC on the same evening of the procedure.

OAC was maintained for at least 3 months after the procedure and was subsequently stopped in patients with a CHA_2_DS_2_-VASC-score of 0 or 1 (in case of female gender). In patients with CHA_2_DS_2_-VASC-score above these values, OAC was continued.

AADs were continued or prescribed for at least three months following CA during the blanking period. Further, all patients were treated with a proton pump inhibitor for 6 weeks after the procedure to reduce the risk of atrioesophageal fistula.

### Follow-up

FU visits were scheduled at 6, 12, and 24 months after the procedure, and included laboratory tests, transthoracic echocardiography, and a 12-lead electrogram (ECG). During each visit, we assessed the clinical status, heart rhythm, medication, and occurrence of a procedure or therapy-related complications. Heart rhythm was further assessed via ambulatory 24-h Holter monitoring at three and then every 6 months after the ablation. Additional information in the KT group was obtained from FU nephrological visits.

### Study objectives

The primary safety end point was a composite of procedure-related complications. These included vascular access complications (arteriovenous fistula, pseudoaneurysm, or hematoma that required intervention), phrenic nerve injury, cardiac tamponade, stroke or transient ischemic attack (TIA), procedure-related kidney injury (an acute kidney injury (AKI) or onset/progression of CKD, defined according to KIDGO guidelines [12], with a causal relationship with the ablation procedure), sedation-related complications, PV stenosis, atrioesophageal fistula, and death.

The secondary endpoints were freedom from AF at FU and impact of the renal function after the procedure and at the end of FU.

### Statistics

Statistical analysis was performed using the SPSS software (IBM SPSS Statistics, version 28). The CG was generated using the propensity score (PS) matching function. We selected four covariates to perform PS: age, gender, type of AF (paroxysmal, persistent, long-standing persistent), and ablation technique (CBA, RFA and LBA). Then, we used a 1:3 nearest neighbor matching method, without replacement and with a 0.005 caliber. Categorical variables were reported as absolute and relative frequencies and were compared using the chi-square or Fischer’s exact test, as appropriate. Continuous variables were tested for normal distribution using the Shapiro–Wilk test and were reported as mean ± standard deviation (SD) if normally distributed or as median with interquartile range (first quartile, third quartile) if skewed distributed. Subsequently, we compared continuous variables using Student’s *t*-test, if normally distributed, or with the corresponding nonparametric tests (Mann–Whitney *U* test or Wilcoxon signed-rank test) if they showed a skewed distribution. The arrhythmia-free survival rate was estimated using the Kaplan–Meier method, and the two groups were compared using the log rank test. All *p*-values are 2-sided, and a *p*-value < 0.05 was considered significant.

## Results

### Patient population

Baseline characteristics are summarized in Table 1. Sixty-four patients were analyzed, 16 in the KT group and 48 in the CG. Patients in the KT group had significantly higher baseline creatinine levels (180 µmol/l vs. 89.5 µmol/l, *p* < 0.001) and a lower eGFR (32 ml/min/1.73 m^2^ vs. 74 ml/min/1.73 m^2^, *p* < 0.001).

**Table 1:** Patients baseline characteristics

		Kidney transplantation *n* = 16	Control *n* = 48	*P*
Age, years		59.5 ± 11.34	60.35 ± 10.26	0.780
Male gender, *n* (%)		10/16 (62.5%)	30/48 (62.5%)	1
Left ventricular ejection fraction, %		55.13 ± 6.88	52.31 ± 9.61	0.187
Structural heart disease, *n* (%)		6/16 (37.5%)	10/48 (20.8%)	0.199
Ischemic heart disease, *n* (%)		4/16 (25%)	10/48 (20.8%)	0.736
Heart failure, *n* (%)		2/16 (12.5%)	8/48 (16.7%)	1
Arterial hypertension, *n* (%)		13/16 (81.3%)	31/48 (64.6%)	0.351
Diabetes, *n* (%)		4/16 (25%)	4/48 (8.3%)	0.099
Vascular disease, *n* (%)		6/16 (37.5%)	6/48 (12.5%)	0.058
Liver failure, *n* (%)		0/16 (0%)	0/48 (0%)	1
CKD, *n* (%)		16/16 (100%)	6/48 (12.5%)	< 0.001
CKD stage	≤ II (GFR ≥ 60), *n* (%)	0/16 (0%)	35/48 (72.9%)	< 0.001
	III (GFR = 30–59), *n* (%)	10/16 (62.5%)	11/48 (22.9%)	0.005
	≥ IV (GFR < 30), *n* (%)	6/16 (37.5%)	2/48 (4.2%)	0.002
Baseline creatinine level, mg/dL		180 (150.3, 202.3)	89.5 (78.3, 108.3)	< 0.001
Baseline-eGFR, mL/min/1.73 mq		32 (23.8, 40.5)	74 (55, 88.3)	< 0.001
Ischemic stroke, *n* (%)		0/16 (0%)	3/48 (6.3%)	0.567
TIA, *n* (%)		2/16 (12.5%)	1/48 (2.1%)	0.156
NYHA	I, *n* (%)	10/16 (62.5%)	22/48 (45.8%)	
	II, *n* (%)	3/16 (18.8%)	16/48 (33.3%)	
	III, *n* (%)	0/16 (0%)	1/48 (2.1%)	
CHADS-VASC	0, *n* (%)	1/16 (6.3%)	24/48 (50%)	
	1, *n* (%)	1/16 (6.3%)	7/48 (14.6%)	
	2, *n* (%)	6/16 (37.5%)	11/48 (22.9%)	
	3, *n* (%)	3/16 (18.8%)	2/48 (4.2%)	
	≥ 4, *n* (%)	5/16 (31.2%)	4/48 (8.3%)	
INR value		1.25 (1.09, 1.45)	1.19 (1, 1.56)	0.710
Platelets/mm^3^		236.38 ± 41.83	214.69 ± 45.26	0.096
Hemoglobin, g/dL		11.75 (10.77, 12.42)	14.35 (13.2, 15.07)	< 0.001
Rhythm	SR, *n* (%)	11/16 (68.8%)	28/48 (58.3%)	0.561
	Atrial flutter, *n* (%)	3/16 (18.8%)	5/48 (10.4%)	0.401
	Atrial fibrillation, *n* (%)	2/16 (12.5%)	10/48 (20.8%)	0.714
	AT, *n* (%)	0/16 (0%)	5/48 (10.4%)	0.319
AF	PAF, *n* (%)	8/16 (50%)	16/48 (33.3%)	0.250
	Persistent, *n* (%)	7/16 (43.8%)	31/48 (64.6%)	0.156
	Long-standing, *n* (%)	1/16 (6.3%)	1/48 (2.1%)	0.440
AAD at baseline, *n* (%)		9/16 (56.3%)	18/48 (37.5%)	0.246
Anticoagulation before	None, *n* (%)	2/16 (12.5%)	10/48 (20.8%)	0.714
	NOAC, *n* (%)	12/16 (75%)	34/48 (70.8%)	1
	VKA, *n* (%)	2/16 (12.5%)	4/48 (8.3%)	0.620
LA dilatation, *n* (%)		8/16 (50%)	30/47 (63.8%)	0.383
LA volume, ml/m^2^		50.63 ± 21.36	30.70 ± 11.09	< 0.001
Hospitalization, days		3 (2.25, 4)	3 (2, 4)	0.177

*CKD* chronic kidney disease, *eGFR* estimated glomerular filtration rate, *TIA* transient ischemic attack, *NYHA* New York Heart Association, *INR* international normalized ratio, *SR* sinus rhythm, *AF* atrial fibrillation, *PAF* paroxysmal atrial fibrillation, *AAD* antiarrhythmic drug, *NOAC* non-vitamin K antagonist oral anticoagulant, *VKA* vitamin K antagonist, *LA* left atrium

We found no statistically significant difference between the groups regarding the type of AF. In the KT group and CG, 50% and 33.3% had paroxysmal AF (*p* = 0.25), 43.8% and 64.6% had persistent AF (*p* = 0.15), and 6.3% and 2.1% had long-standing persistent AF (*p* = 0.44), respectively.

The mean ejection fraction (EF) was also similar (55.1% vs. 52.3%, *p* = 0.18).

Regarding thromboembolic risk, the KT group showed a significantly higher CHA2DS2-VASc score than the CG (*p* < 0.01).

A percentage of 56.3 of patients in the KT group and 37.5% in the CG (*p* = 0.24) were on AAD therapy before the ablation. The use of OAC was homogeneous between study groups. A percentage of 75 of patients in the KT group and 70.8% in the CG (*p* = 1.00) were on NOAC therapy before the procedure, whereas 12.5% in the KT group and 8.3% in the CG (*p* = 0.62) were on VKA.

### Procedural data and primary safety endpoint

Procedural data are summarized in Table 2.

**Table 2:** Procedural data and periprocedural complications

	Kidney transplantation *n* = 16	Control *n* = 48	*p*
Procedure duration, min	122.5 (62.5, 191)	83 (60, 135)	0.141
Contrast, ml	30 (10, 55)	50 (40, 75)	0.02
Type of procedure			
Cryoballoon	3/16 (18.8%)	13/48 (27.1%)	0.740
RF	12/16 (75%)	22/48 (45.8%)	0.050
Laser	1/16 (6.3%)	13/48 (27.1%)	0.159
RF duration, sec	1874 ± 1272.5	1004.9 ± 850.3	0.102
Procedures with no contrast, *n* (%)	7/16 (43.7%)	0/48 (0%)	< 0.001
Additional lesion			
No	12/16 (75%)	36/48 (75%)	1
Roof line	3/16 (18.8%)	0/48 (0%)	0.013
Posterior line	1/16 (6.3%)	0/48 (0%)	0.250
Others	0/16 (0%)	12/48 (25%)	0.028
Total cryoablation time, sec	498.3 ± 210.6	782.2 ± 204.3	0.020
Postoperative creatinine level	180.5 (152.3, 223)	88.5 (78, 108)	< 0.001
Postoperative eGFR	32 (26.3, 39.8)	72.5 (59.5, 86.5)	< 0.001
Periprocedural complications, *n* (%)	1/16 (6.3%)	3/48 (6.3%)	1
Death	0/16 (0%)	0/48 (0%)	
Atrioesophageal fistula	0/16 (0%)	0/48 (0%)	
Stroke/TIA	0/16 (0%)	0/48 (0%)	
Major periprocedural bleeding	1/16 (6.3%)	0/48 (0%)	
Pericardial effusion /tamponade	0/16 (0%)	0/48 (0%)	
Artero-venous fistula or pseudoaneurysm	0/16 (0%)	0/48 (0%)	
Procedure related kidney injury	1/16 (6,3%)	0/48 (0%)	
PV stenosis	0/16 (0%)	0/48 (0%)	
Sedation related complication	0/16 (0%)	1/48 (4.2%)	
Phrenic nerve palsy in patients who underwent CB and LB ablation	0/4 (0%)	2/26 (7.6%)	
AAD post-ablation, *n* (%)	8/16 (50%)	40/48 (83.3%)	0.585
Anticoagulation After			
None, *n* (%)	1/16 (6.3%)	0/48 (0%)	0.250
NOAC, *n* (%)	13/16 (81.3%)	44/48 (91.7%)	0.353
VKA, *n* (%)	2/16 (12.5%)	4/48 (8.3%)	0.634

*RF* radiofrequency, *eGFR* estimated glomerular filtration rate, *TIA* transient ischemic attack, *PV* pulmonary vein, *CB* cryo balloon, *LB* laser balloon, *AAD* antiarrhythmic drug, *NOAC* non-vitamin K antagonist oral anticoagulant, *VKA* vitamin K antagonist

RFA was the most commonly performed procedure for PVI, used in 12/16 (75%) patients in the KT group and 22/48 (45.8%) in the CG. CBA was performed in 3/16 (18.8%) patients in the KT group and 13/48 (27.1%) in the CG, and LBA was performed in 1/16 (6.3%) patients in the KT group and 13/48 (27.1%) in the CG.

Procedural time did not differ between groups (122.5 min in KT group vs. 83 min in CG, *p* = 0.14). The use of intravenous contrast was significantly lower in the KT group (30 ml vs. 50 ml, *p* = 0.02). No contrast was used in seven patients in the KT group.

There was no difference between the study groups regarding the primary safety endpoint (6.3% vs. 6.3%, *p* = 1.00). Nonetheless, one patient in the KT experienced retroperitoneal bleeding from the external iliac vein, which required multiple blood transfusions and a prolonged hospital stay. In the CG, two patients experienced transient phrenic nerve paralysis, which recovered at follow-up, and one developed procedure-related aspiration pneumonia. In both cohorts, no cerebral ischemia, atrioesophageal fistula, or death occurred.

**Fig. 1:**
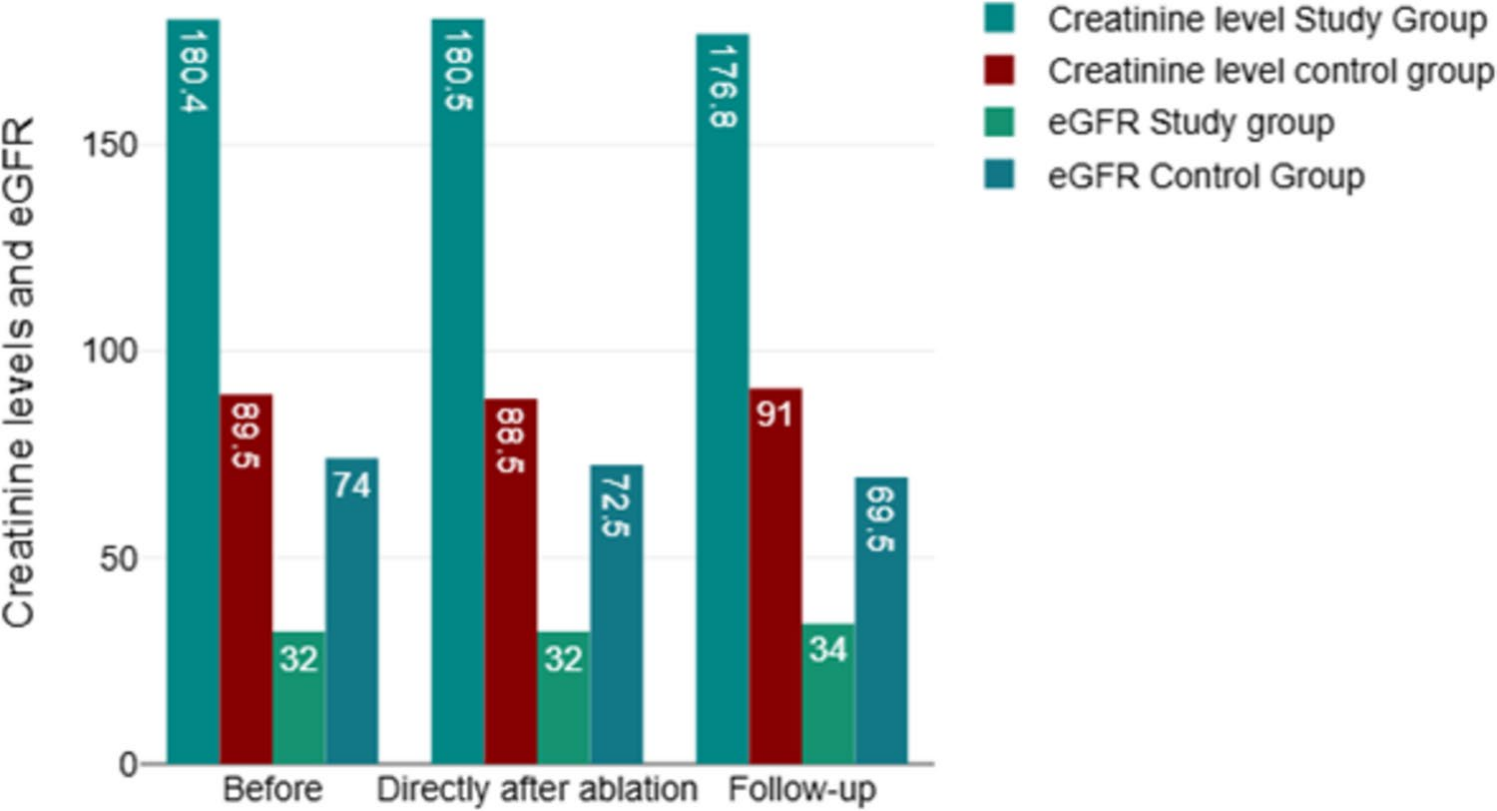
Kidney function before, directly after ablation and at last follow-up. The ablation procedure did not significantly impact the renal function estimated with the eGFR in the KT group (32 ml/min/1.73 m^2^ before the procedure vs. 34 ml/min/1.73 m.^2^ at the end of FU, *p* = 0.93)

Finally, the median eGFR did not change significantly after the ablation procedure in both study groups (Fig. 1).

### Follow-up and secondary endpoints

The mean FU duration was 17 months in the KT group and 15 months in the CG (*p* = 0.70).

**Fig. 2:**
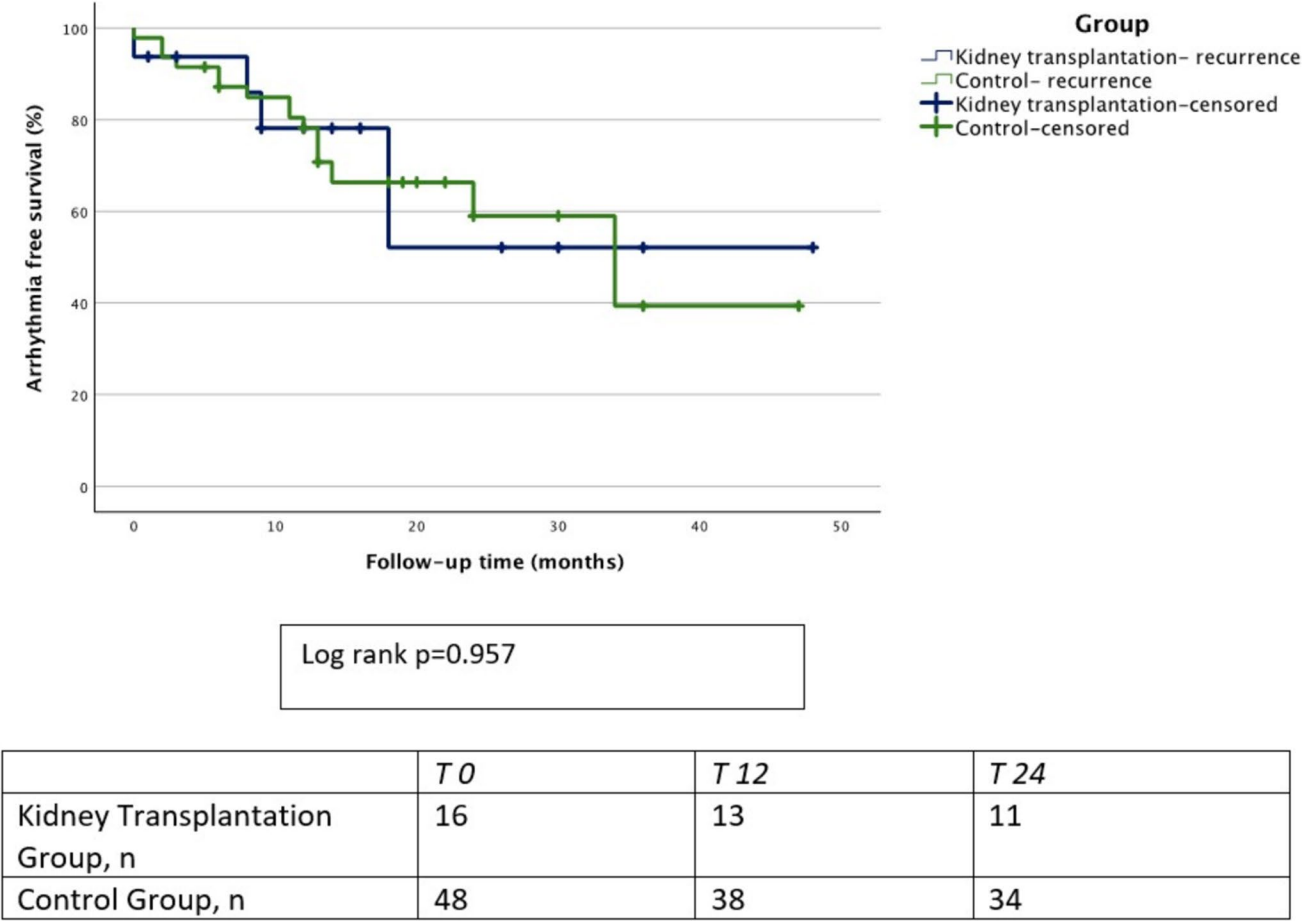
Kaplan–Meier arrhythmia free survival curves and number at risk table: the Kaplan–Meier estimated arrhythmia-free survival during follow-up was 69% (95% confidence interval [CI] 57.7–80.3%) for the study group and 70.1% (95% CI 63.2–77.1%) for the control group (log rank *p* = 0.95). The 12-month arrhythmia-free survival was 81.3% for the study group and 79.2% for the control group (*p* = 1)

We found no difference regarding arrhythmia recurrences during FU. The 12-month arrhythmia-free survival rate was 81.3% in the KT group and 79.2% in the CG (*p* = 1.00). The total estimated arrhythmia-free survival rate, calculated by the Kaplan–Meier method, was 69% (95% confidence interval [CI] 57.7–80.3%) in the KT group and 70.1% (95% CI 63.2–77.1%) in the CG (log-rank *p* = 0.95) (Fig. 2). A percentage of 68.8 of the patients were free of arrhythmia at the last FU in both groups.

A second ablation procedure was performed in 11 patients: three (18.8%) in the KT group and eight (17%) in the CG (*p* = 1.00) (Table 3).

**Table 3:** Follow-up data

	Kidney transplantation *n* = 16	Control *n* = 48	*p*
Follow-up duration, months	17 (9.8, 33.8)	15 (12, 24)	0.707
Arrhythmia-free survival	11/16 (68.8%)	33/48 (68.8%)	1
Arrhythmia-free survival, 1 year	13/16 (81.3)	38/48 (79.2%)	1
AAD at follow-up, *n* (%)	0/16 (0%)	9/46 (19.6%)	0.096
Anticoagulation at follow-up			
None, *n* (%)	2/16 (12.5%)	11/46 (23.9%)	0.483
SAPT, *n* (%)	1/16 (6.3%)	3/46 (6.5%)	1
NOAC, *n* (%)	11/16 (68.8%)	29/46 (63%)	0.768
VKA, *n* (%)	2/16 (12.5%)	3/46 (6.5%)	0.596
INR	1.20 (0.95, 1.64)	1.25 (1.1, 1.52)	0.317
Creatinine level	179.5 (168.8, 208.3)	92 (80, 108)	< 0.001
eGFR	34 (28.3, 38)	70 (59, 88.5)	< 0.001
Re-ablation during FU	3/16 (18.8%)	8/47 (17%)	1

*AAD* antiarrhythmic drug, *SAPT* single antiplatelet therapy, *NOAC* non-vitamin K antagonist oral anticoagulant, *VKA* vitamin K antagonist, *INR* international normalized ratio, *eGFR* estimated glomerular filtration rate, *CKD* chronic kidney disease, *FU* follow-up

All KT patients were no longer receiving AADs at the last follow-up visit, while in the CG, 19.6% of patients were still on AAD therapy (*p* = 0.09). Two KT patients discontinued their AADs (amiodarone and sotalol) during the blanking period due to QT prolongation. Both patients had cyclosporine in their immunosuppressive regimen. No torsade de pointes or polymorphic ventricular tachycardia was observed during follow-up.

The ablation procedure did not significantly impact the renal function estimated with the eGFR in the KT group (32 ml/min/1.73 m^2^ before the procedure vs 34 ml/min/1.73 m^2^ at the end of FU, *p* = 0.93) (Fig. 1).

The patient in the KT group, who had a prolonged hospital stay because of a major bleeding complication, developed an AKI and showed a further decrease in kidney function at follow-up, which ultimately led to the loss of the graft and the need for dialysis.

One patient in the KT group died during FU due to non-CV causes.

## Discussion

The aim of this study, which comprises the largest cohort of KT patients who underwent AF ablation, was to analyze the safety, efficacy, and impact on kidney function of CA of AF in this population, comparing it with a matched cohort.

### Primary endpoint: safety

In our study, the overall complication rate in KT patients undergoing CA of AF was not higher than in a CG.

CKD patients, including KT recipients, show a procoagulant state [13], lower GFR values, and a consequent need for a reduced dose of OAC. This leads to an increased risk of LAA thrombus formation [14] and a higher incidence of thromboembolic events [15, 16].

Further, patients with advanced CKD or renal transplantation show a bleeding diathesis [13], difficult and heterogeneous OAC management [17, 18], and a high prevalence of peripheral artery disease [19], which are risk factors for hemorrhagic complications. Previous studies have already shown that patients with advanced kidney disease undergoing LAA occlusion and CA of AF have a higher frequency of bleeding complications, including vascular access complications (VAC) [20, 21].

However, in a single-center study by Su et al., none of the nine KT patients who underwent CA of AF suffered major periprocedural complications [22]. Our study confirmed the safety of CA in KT. Nevertheless, the complications in the control group recovered at follow-up and did not affect the long-term quality of life of the patients. Conversely, one patient in the KT group experienced post-procedural major bleeding at the vascular access site. This patient had an eGFR of 20 ml/min/1.73 m^2^ before ablation and was receiving low-dose apixaban. This patient developed a retroperitoneal hematoma and AKI during the following hospital stay, with a further decrease in kidney function at follow-up, which ultimately led to the loss of the graft and the need for dialysis.

Thus, our study emphasizes the importance of careful OAC management, peri-interventional anticoagulation, and VAC in this patient’s cohort.

### Secondary endpoint: atrial fibrillation recurrence rate

Even if impaired renal function is a known risk factor for AF recurrences after CA, and the recurrence rate is proportionally higher with lower values of eGFR or in patients undergoing dialysis [23–26], our study showed in a population that included only KT patients, that CA had a recurrence rate and a need for a redo procedure similar to a matched cohort.

The efficacy of CA in this population was already suggested in a previous study, that found no difference in the efficacy rate of AF ablation between solid organ transplant recipients, including KT patients, and a matched cohort [22].

These results are of particular importance since, according to a study from Potpara et al., cardiologists tend to suggest less frequently a CA of AF if creatinine clearance is below 30 mL/min [18].

Notably, no KT patient was receiving AADs at the end of follow-up, whereas 19.6% of the CG were still on antiarrhythmic therapy. Half of the transplanted patients were treated with an AAD at study inclusion. During follow-up, two of them discontinued their antiarrhythmic therapy because of QT prolongation. The use of AADs after KT is challenging. A higher prevalence of CV diseases in these patients [2] makes AAD selection difficult. Moreover, KT patients under immunosuppressive therapy show a prolongation of the QT-interval [27]. Finally, amiodarone and dronedarone interact with tacrolimus and cyclosporine by competing for the cytochrome (CYP) P450 [28–32].

Therefore, CA has a specific positive therapeutic impact in this population since it may allow interruption of AADs, preventing side effects and interactions with immunosuppressive therapy.

### Secondary end point: kidney function

In our KT patients’ cohort, the median GFR did not deteriorate acutely after ablation, showing a slight increase during FU without reaching statistical significance (Fig. 1).

AF is associated with a deterioration of kidney function [33]. Previous studies have suggested that CA may prevent the decrease of eGFR in patients with CKD, especially in those with reduced AF burden after the procedure [34, 35].

Nevertheless, CA could pose a risk of AKI because of periprocedural complications and the use of contrast to perform transseptal puncture and angiography of the pulmonary veins.

In fact, the patient in the KT group, who experienced post-ablation major bleeding, showed an AKI during the following hospital stay. The same patient had a further decrease in kidney function at follow-up, which ultimately led to the loss of the graft and the need for dialysis.

Preservation of kidney function in KT patients is paramount. A low CA-related complication rate and limited or no use of contrast during the procedure could allow a reduced rate of periprocedural AKI, and a lower atrial fibrillation burden after the procedure could help preserve kidney function at follow-up.

Among single-shot devices, the LBA technique could provide a potential advantage over CBA since it could eliminate the need for contrast while maintaining fast procedural times and efficacy [36].

Our study did not include the use of PFA to treat these patients. Even though PFA proved an effective and safe technique for CA of AF, its impact on renal function remains to be clarified, since cases of AKI from hemoglobinuria after the procedure have been described [37, 38].

### Study limitations

We conducted a non-randomized observational study based on a small population, which could hinder the generalization of the results. Further, the small population size did not allow us to assess hard clinical endpoints at follow-up, such as major adverse cardiovascular events. Finally, the registry size used to obtain the CG did not allow us to compensate for some differences between populations through the propensity score matching function. Additionally, logistical constraints prevented us from obtaining the control group from both centers, which might have resolved this issue. However, although the two groups differed in some features, ablation proved equally safe and effective in both.

The enrolled patients did not receive continuous ECG monitoring at follow-up, which may have led to an underestimation of AF recurrences at FU.

Selection bias cannot be excluded since we included only KT patients referred to the CA enrolling centers. Finally, patients were treated in centers with a high volume of CA procedures, and our results could not be reproducible in low-volume centers.

## Conclusions

In our patient cohort, CA was effective in treating AF in KT patients. Moreover, it did not negatively impact kidney function and allowed AAD discontinuation, reducing the risk of side effects and interactions with immunosuppressive therapy. Procedure-related complications remain relevant since they may affect graft function in this subset of patients.

## Supplementary Information

Below is the link to the electronic supplementary material.

Supplementary file1 (DOCX 14 KB)

## Abbreviations

ACT: Activated clotting time
AAD: Antiarrhythmic drug
AF: Atrial fibrillation
AKI: Acute kidney injury
CA: Catheter ablation
CAFE: Complex fractionated electrograms
CB: Cryoballoon
CG: Control group
CKD: Chronic kidney disease
CV: Cardiovascular
DOAC: Direct oral anticoagulant
ECG: Electrocardiogram
ESRD: End-stage renal disease
FU: Follow-up
eGFR: Estimated glomerular filtration rate
KDIGO: Kidney Disease Improving Global Outcomes
KT: Kidney transplantation
LAA: Left atrial appendage
LBA: Laser balloon ablation
INR: International normalized ratio
PV: Pulmonary vein
PVI: Pulmonary vein isolation
RFA: Radiofrequency ablation
SD: Standard deviation
TIA: Transient ischemic attack
OAC: Oral anticoagulation
VAC: Vascular access complications
VKA: Vitamin K antagonist
