# Supplementary material for: Safety and efficacy of atrial fibrillation ablation in kidney transplant patients
Source: J Interv Card Electrophysiol. 2025 Feb 28;68(5):1017–26. doi: 10.1007/s10840-025-02006-x (PMC12317879; doi:10.1007/s10840-025-02006-x)
Supplement: Supplementary file 1 — Supplementary file1 (DOCX 14 KB) [file 10840_2025_2006_MOESM1_ESM.docx]

**Atrial fibrillation ablation techniques used in the study**

**Cryoballoon ablation:**

Esophageal temperature monitoring was conducted with an esophageal temperature probe (Sensitherm; St Jude Medical, Inc. or Circa S‐Cath™; Circa Scientific). After the transseptal puncture (TSP) and pulmonary vein (PV) selective angiography, the transseptal sheath was exchanged for the 15F Flexcath Advance sheath. A specific balloon (second-generation Arctic Front Advance®; Medtronic, Inc.) was advanced in the left atrium (LA) and placed at the antrum of the PVs, where a single, circumferential lesion was obtained through cooling of the distal section of the device at a temperature of around -50 °C for 180 seconds. Complete occlusion of PV by cryoballoon (CB) was verified by contrast injections inside the PV. Contrast leakage in the LA marked incomplete occlusion, while retention of contrast medium inside the PV indicated total occlusion. A circular mapping catheter (CMP) on the tip of the CB was used to confirm PVI during the delivery of the lesion. If we detected an incomplete occlusion that could not be corrected after mobilization of the catheter, lesion delivery was interrupted, and a new 180-second freeze cycle was started. New lesion delivery was also started if PVI was not achieved after the first freeze. During right-side PV isolation, phrenic nerve pacing was performed at maximum output with a cycle duration of 1000-1200 milliseconds using a diagnostic catheter (7F, Webster TM, Biosense Webster, Inc) located in the superior vena cava. The operator used tactile feedback and a fluoroscopic view of diaphragmatic contraction to confirm phrenic nerve capture. If weakening of diaphragmatic contraction was noted, energy delivery was interrupted.

**Radiofrequency pulmonary vein isolation:**

In radiofrequency (RF) ablation, after TSP and selective PV angiographies, we performed 3D mapping of the left atrium using a circular mapping catheter (CMP) (LASSO™ Catheter; Biosense Webster, Diamond Bar, CA) and an electroanatomical mapping system (CARTO; Biosense Webster, Diamond Bar, CA). PV angiography and local electrical signals were used to define the PV ostia. PVI was conducted using an irrigated radiofrequency catheter with a 3.5 mm tip (NaviStar ThermoCool; Biosense Webster). During energy delivery, the CMP was placed inside each PV ostium to record electric PV potentials and to guide PVI. At the end of ablation, after a 30-minute waiting period, the procedural endpoint for PVI was defined as the presence of an entrance and exit block for each PV recorded through the CMP.

**Laser Ablation:**

In laser balloon ablation (LBA), a double transeptal puncture was performed. The laser balloon (LB) was advanced into the LA after replacing the transseptal sheath with a 12-Fr steerable sheath (CardioFocus) over a guidewire. PV ostia were identified using selective pulmonary venography. To perform PVI, we used the third generation LB system (LB3; HeartLight X3), which introduced a motor control system that enables uninterrupted, high-speed, circumferential lesion at 13 W. Whenever the use of this system is not possible, the LB3 also allows for point-by-point ablation in a similar manner to RF ablation. The ablation lines were delivered in a figure-eight pattern to isolate both ipsilateral PV ostia by interconnecting two incomplete semicircular ablation lines at the PV carina.

During right-side PV isolation, we performed phrenic nerve pacing using a diagnostic catheter (7F, Webster TM, Biosense Webster, Inc) in the superior vena cava to detect phrenic nerve injury.

The operator used tactile feedback and a fluoroscopic view of diaphragmatic contraction to confirm phrenic nerve capture. If weakening of diaphragmatic contraction was noted, energy delivery was interrupted.

At the end of the procedure, we used the second TSP to insert a 15-mm CMP (Lasso, Biosense Webster) into each PV ostia to check for PVI. Additional lesions were performed in the case of a non-isolated pulmonary vein.
